# Genomic analysis reveals that *Pseudomonas aeruginosa *virulence is combinatorial

**DOI:** 10.1186/gb-2006-7-10-r90

**Published:** 2006-10-12

**Authors:** Daniel G Lee, Jonathan M Urbach, Gang Wu, Nicole T Liberati, Rhonda L Feinbaum, Sachiko Miyata, Lenard T Diggins, Jianxin He, Maude Saucier, Eric Déziel, Lisa Friedman, Li Li, George Grills, Kate Montgomery, Raju Kucherlapati, Laurence G Rahme, Frederick M Ausubel

**Affiliations:** 1 Department of Molecular Biology, Massachusetts General Hospital, Cambridge Street, Boston, Massachusetts, 02114, USA; 2 Department of Genetics, Harvard Medical School, Avenue Louis Pasteur, Boston, Massachusetts, 02115, USA; 3 Current address: Microbia, Inc., Bent Street, Cambridge, Massachusetts, 02141, USA; 4 Envivo Pharmaceuticals, Inc., Arsenal Street, Watertown, Massachusetts, 02472, USA; 5 Department of Microbiology and Molecular Genetics, Harvard Medical School, Longwood Avenue, Boston, Massachusetts, 02115. USA; 6 Department of Surgery, Massachusetts General Hospital, Fruit Street, Boston, Massachusetts, 02114, USA; 7 Current address: Université de Montréal, Station Centre-ville, Montréal, H3C 3J7, Canada; 8 Current address: INRS-Institut Armand-Frappier, boul. des Prairies, Laval, Quebec, H7V 1B7, Canada; 9 Current address: Cubist Pharmaceuticals, Inc., Hayden Avenue, Lexington, Massachusetts, 02421, USA; 10 Harvard Medical School - Partners Healthcare Center for Genetics and Genomics, Landsdowne Street, Cambridge, Massachusetts, 02139, USA; 11 Current address: Core Facilities, Cornell University, Thurston Avenue, Ithaca, New York, 14850, USA

## Abstract

**Background:**

*Pseudomonas aeruginosa *is a ubiquitous environmental bacterium and an important opportunistic human pathogen. Generally, the acquisition of genes in the form of pathogenicity islands distinguishes pathogenic isolates from nonpathogens. We therefore sequenced a highly virulent strain of *P. aeruginosa*, PA14, and compared it with a previously sequenced (and less pathogenic) strain, PAO1, to identify novel virulence genes.

**Results:**

The PA14 and PAO1 genomes are remarkably similar, although PA14 has a slightly larger genome (6.5 megabses [Mb]) than does PAO1 (6.3 Mb). We identified 58 PA14 gene clusters that are absent in PAO1 to determine which of these genes, if any, contribute to its enhanced virulence in a *Caenorhabditis elegans *pathogenicity model. First, we tested 18 additional diverse strains in the *C. elegans *model and observed a wide range of pathogenic potential; however, genotyping these strains using a custom microarray showed that the presence of PA14 genes that are absent in PAO1 did not correlate with the virulence of these strains. Second, we utilized a full-genome nonredundant mutant library of PA14 to identify five genes (absent in PAO1) required for *C. elegans *killing. Surprisingly, although these five genes are present in many other *P. aeruginosa *strains, they do not correlate with virulence in *C. elegans*.

**Conclusion:**

Genes required for pathogenicity in one strain of *P. aeruginosa *are neither required for nor predictive of virulence in other strains. We therefore propose that virulence in this organism is both multifactorial and combinatorial, the result of a pool of pathogenicity-related genes that interact in various combinations in different genetic backgrounds.

## Abstract 2

Sequencing of a highly virulent strain of *Pseudomonas aeruginosa *and comparison to a previously sequenced, less pathogenic, strain, together with experimental testing in a *C. elegans model*, suggests that *Pseudomonas *virulence is multifactorial and combinatorial.

## Background

The potential virulence of bacterial pathogens is significantly modulated by the presence of pathogenicity islands [1,2], which are clusters of one or more virulence-related genes that are often acquired by horizontal gene transfer. The introduction of these virulence islands can allow a previously nonvirulent isolate to infect a particular host. Commonly, this switch to a simpler and more stable environment within a host (as opposed to the more complex outside environment) is followed by gene loss and genome reduction that improve the ability of the pathogen to survive in the host but also restrict the range of hosts available to the bacterium [3,4]. In contrast, free-living bacteria that dominate in complex environments (such as soil) tend to have genomes that continue to acquire DNA and undergo expansion rather than reduction.

*Pseudomonas aeruginosa*, a ubiquitous Gram-negative soil organism, is an important opportunistic human pathogen that infects injured, burned, immunodeficient, and immunocompromised patients, and causes persistent respiratory infections in individuals suffering from cystic fibrosis (CF) [5,6]. The genome sequence of the widely studied *P. aeruginosa *strain PAO1 (originally a wound isolate) revealed that it possesses a large number of genes that are involved in regulation, catabolism, transport, and efflux of organic compounds, as well as several putative chemotaxis systems [7], all of which potentially contribute to the remarkable ability of this bacterium to adapt to a wide range of environmental niches.

Different *P. aeruginosa *isolates share a remarkable amount of similarity in their genomes. When DNA derived from several *P. aeruginosa *strains was hybridized to a PAO1 microarray, between 89% and 98% of the PAO1 sequences were detected [8,9]. Whole-genome shotgun sequencing of two CF isolates and one environmental strain revealed that, aside from this apparent highly conserved core set of *P. aeruginosa *genes, differences were largely due to strain-specific islands of genes, consisting either of genes with similar or related function but divergent DNA sequences (such as genes for biosynthesis of the O-antigen component of lipopolysaccharide, genes for flagellar biosynthesis, or alternate forms of genes for the bacteriocidal pyocins) or genes that are entirely absent in some strains [10].

Despite the overall genome similarity among diverse *P. aeruginosa *strains, differences in complex phenotypes such as pathogenicity can be striking. For example, the clinical isolate PA14 is significantly more virulent than PAO1 in a wide range of hosts, including mice, the nematode *Caenorhabditis elegans*, the insect *Galleria mellonella*, and the plant *Arabidopsis thaliana *[11-13]. PA14 genes required for full virulence include genes common to many if not all *P. aeruginosa *strains, including global transcriptional regulators such as *gacA*; genes that are involved in pathogenesis-related processes such as motility, quorum sensing, and phenazine biosynthesis; and genes that encode secreted cellulytic factors and toxins such as *ExoU*, exotoxin A, phospholipase C, and elastase [12-18]. On the other hand, novel PA14 genes that are absent in PAO1 (and potentially absent in other isolates) have also been identified as being required for pathogenicity in model hosts and mice [11,15,17,18], and at least some of these genes appear to reside on large pathogenicity islands [19]. Taken together, these studies suggest that PA14 pathogenicity is multifactorial, requiring the cumulative (and potentially coordinated) action of multiple virulence factors, some of which are components of the basic core genome, whereas others are located on classically defined virulence islands.

The experiments described in this paper were designed to test the hypothesis that the enhanced virulence of PA14 compared with PAO1 is mostly a consequence of recognizable pathogenicity (virulence) islands that are present in PA14 but absent in PAO1. To expand our tools for dissecting *P. aeruginosa *virulence and to test the hypothesis that strain differences in virulence are due to the acquisition of strain-specific genes, we sequenced the PA14 genome and performed a functional analysis of genes that are present in PA14 but absent in PAO1 to assess their contribution to pathogenicity.

## Results and discussion

### PA14 genome sequence

To identify all of the putative pathogenicity islands that distinguish PA14 from PAO1, we sequenced the PA14 genome and found that it contains a slightly larger chromosome (6.5 megabases [Mb] versus 6.3 Mb for PAO1; genome sequence and annotations are available at the Ausubel lab PA14 sequencing website [20] and have also been deposited in GenBank [GenBank: CP000438]). Consistent with previous observations that overall strain similarity is high, we found that approximately 91.7% of the PA14 genome is present in PAO1, and that 95.8% of the PAO1 genome is present in PA14 (Table 1).

**Figure 1:**
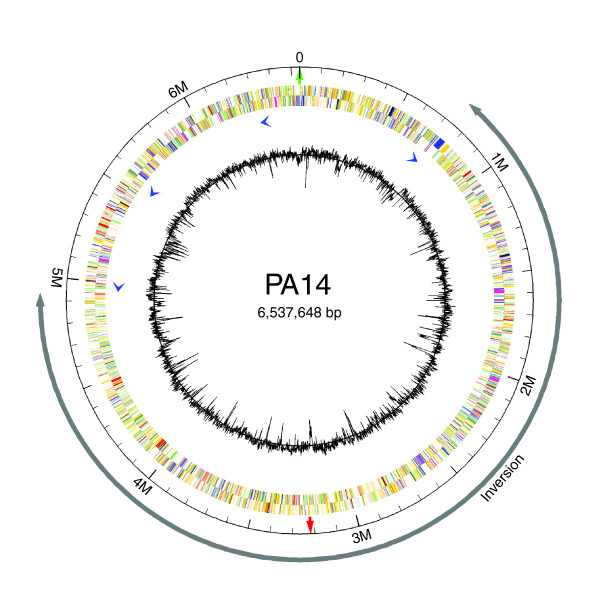
Circular map of the PA14 genome. The outermost circle represents the chromosomal location: major and minor ticks represent 500 and 100 kb increments, respectively. The origin and presumptive terminus of replication are indicated by green and red arrows, respectively. The locations and orientations of predicted genes are shown by rectangles in the next pair of circles; genes on the outer circle are transcribed on the plus strand and genes on the inner circle are transcribed on the minus strand. The genes are color-coded according to functional categories (see below). Blue arrow heads indicated the locations and relative orientations of four ribosomal RNA gene clusters; the published PAO1 sequence contains an inversion (gray arrow) with respect to PA14 resulting from a presumptive recombination event between two of the rRNA clusters. The innermost circle represents the GC content as calculated for non-overlapping 1 kb windows of the plus strand. A linear map and full annotations of each gene (including color codes for functional categories) are available at the Ausubel lab PA14 sequencing website [20] and the full sequence and annotations have been deposited in GenBank (GenBank: CP000438).

**Figure 2:**
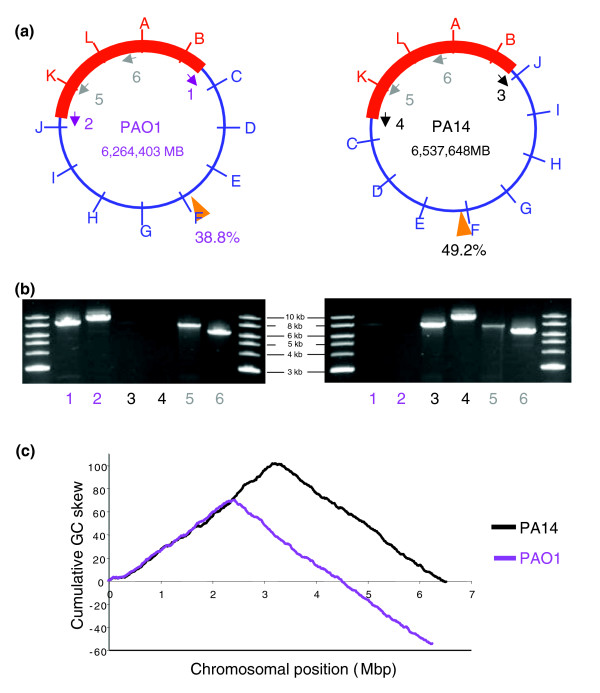
Chromosomal rearrangement in PAO1 repositions the replication terminus relative to the origin. **(a) **Schematic of PAO1 and PA14 chromosomes. The region with the same orientation in both strains is shown with a thick red line; a thin blue line represents the inverted region. Arrows represent the positions and orientations of the four ribosomal RNA clusters. PCR products designed with unique sequences flanking each rRNA cluster are indicated by numbers next to each arrow. PCR products 1 and 2 (purple numbers) are diagnostic for the PAO1 chromosome structure; PCR products 3 and 4 (black numbers) are diagnostic for the PA14 chromosome structure. The position of the presumptive terminus of replication in each strain is indicated by an orange triangle marked with the corresponding position along the chromosome (expressed as the percentage of the whole chromosome, starting from the origin of replication and moving in a clockwise direction). **(b) **Diagnostic long-range PCR spanning each ribosomal RNA repeat demonstrates the inversion in PAO1. PCR products corresponding to the numbers indicated in panel a were generated using genomic DNA from PAO1 (left panel) or PA14 (right panel). PCR products 1 and 2 were obtained only when using PAO1 genomic DNA (the weak background band for product 1 seen using PA14 as a template was also observed for 18 additional *P. aeruginosa *strains tested). PCR products 3 and 4 were obtained only when using PA14 genomic DNA. PCR products 5 and 6 were obtained from both strains. **(c) **GC skew analysis was performed using 1 kb windows, and the cumulative GC skew is shown on the y-axis as a function of chromosomal location (x-axis) for PA14 (black line) and PAO1 (purple line). The position of the peak indicates the likely position of the terminus of replication. For PA14 the peak is centered in the middle of the chromosome (at 49.2% of the genome, between coordinates 3,219,001 and 3,220,000), whereas the peak for PAO1 is offset with respect to the origin of replication as a result of the inversion (occurring at 38.8% of the genome, between coordinates 2,428,001 and 2,429,000). PCR, polymerase chain reaction.

The PA14 and PAO1 genomes are largely colinear with the exception of one major rearrangement that has previously been described: an inversion between two of four dispersed copies of a large ribosomal RNA cluster (Figure 1[7]). We used long-range polymerase chain reaction (PCR) spanning the rRNA clusters to verify the inversion in the sequenced PA14 and PAO1 genomes; PCR products predicted to be indicative of one orientation or the other were only generated when either PAO1 or PA14 DNA was used as the template (Figure 2a,b). When 18 diverse *P. aeruginosa *strains were subjected to this same PCR analysis, all 18 of these isolates were the same or resembled PA14 genome structure with respect to the large inversion, whereas none of the 18 strains had or resembled the PAO1 genome structure (data not shown). Of note, additional PAO1 clones (distinct from the isolate that was sequenced) also appear to contain the same inversion found in PA14 [7].

We also compared the GC skews of the PA14 and PAO1 genomes. GC skew is defined as the value of [G-C]/[G+C] where G and C represent the local base frequencies of G and C, respectively. In prokaryotic genomes there is a bias toward G over C on the leading strand of DNA synthesis [3]. Therefore, when measured at regular intervals along the chromosome (1 kilobase [kb] segments) GC skew tends to have a positive value on the leading strand of DNA synthesis and a negative value on the lagging strand, resulting in polarity changes at the origin and terminus of replication. The putative position of the replicative terminus is therefore operationally defined as the peak of the cumulative GC skew and it typically resides opposite the origin of replication in bacterial genomes [3]. For PA14, the peak GC skew was indeed mapped opposite the replication origin (at 49.2% of the genome; Figure 2a,c). In contrast, the position of the terminus in the sequenced PAO1 chromosome is shifted relative to the origin (at 38.8% of the genome). These observations argue that the PA14 chromosome with respect to this large inversion is more representative of the canonical or ancestral *P. aeruginosa *genome than that of the sequenced PAO1 isolate. This interpretation that is further supported by the finding that the inversion in the sequenced strain occurred sometime after 1990 (a PAO1 cosmid library originally described in that year was shown not to contain the inversion [21]). However, the physiologic consequences of such an inversion and asymmetric replication cycle are not clear.

### PA14 gene annotation

**Figure 3:**
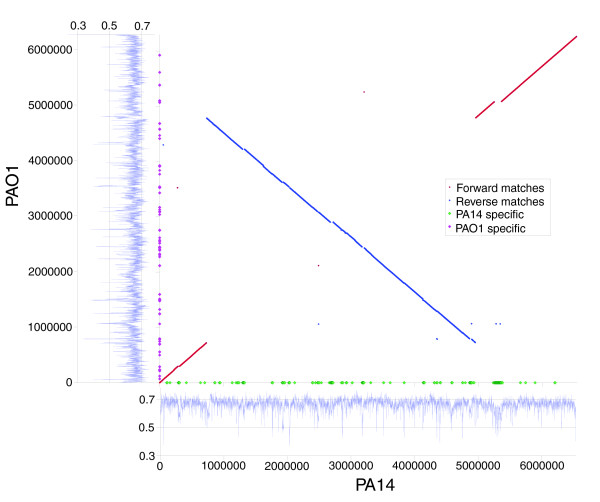
ORF-by-ORF alignments of PA14 and PAO1. Each predicted ORF in PA14 was compared using BLAST with all annotated PAO1 ORFs, and the best match was indicated as a single data point with the chromosomal locations of the match in each genome. Reciprocal BLAST searches were also performed (using individual PAO1 ORFs against the complete set of PA14 ORFs). Matches in the forward direction are indicated by red diamonds, and matches in the reverse orientation are indicated by blue diamonds. Genes that fall outside of the diagonals (non-colinear genes) are a combination of translocations and gene pairs in which a true ortholog is missing in one strain but a similar gene exists elsewhere in its genome and the two genes have been selected as reciprocal best BLAST matches. Genes in one genome that fail to have a BLAST match counterpart in the other genome are represented on the x-axis for PA14-specific genes (green diamonds) and on the y-axis for PAO1-specific genes (pink diamonds). The GC contents for each genome are represented next to the two axes using a light-blue graph; the positions that correspond to GC contents of 30%, 50%, and 70% are indicated. BLAST, basic local alignment search tool; ORF, open reading frame.

We annotated the PA14 genome and identified 5973 predicted open reading frames (ORFs; 322 more than in PAO1; Table 1). Summaries of all predicted PA14 genes and their distribution among different functional categories are presented in Additional data files 2 and 3, respectively. We performed a reciprocal NCBI-BLAST (basic local alignment search tool) search of each gene in either PA14 or PAO1 against the total collection of genes in the other strain (specifically, each PA14 gene was BLASTed against the complete set of PAO1 genes, and *vice versa*). The position of each best BLAST hit was plotted (with the PA14 genomic location on the x-axis, and the PAO1 coordinate on the y-axis; Figure 3), confirming that the two genomes are largely colinear, with the exception of the large inversion described above. Individual PA14 genes that had no counterpart in PAO1 are shown as individual (green) data points on the x-axis, and PAO1 genes absent in PA14 are represented as (pink) data points on the y-axis. Large clusters of these strain-specific genes often correlated with regions of the respective genomes in which the local GC content was lower than that of the total genome (Figure 3).

Using a combination of raw sequence and ORF-based global alignments of the two genomes, we compiled a list of gene clusters present in one strain but absent in the other (58 PA14 regions absent in PAO1 containing 478 genes, and 54 PAO1 regions absent in PA14 containing 234 genes; Table 1 and Additional data file 4). We refer to these as PA14-specific or PAO1-specific regions for the purposes of this discussion (recognizing that these genes may be present in other isolates of *P. aeruginosa *and are not strictly strain specific). Many of these gene clusters have hallmarks of horizontally acquired DNA, including direct repeats, insertion sequences, tRNA genes at their boundaries (data not shown), and/or anomalous GC contents (PA14-specific clusters have an average GC content of 59.6%, which is more than one standard deviation below the whole genome average of 66.3%; Table 1).

These strain-specific regions contain a high proportion of genes of unknown function as compared with the whole genome. Each PA14 (and PAO1) annotation has a confidence score associated with the described gene function [7,22]. A class 1 designation refers to genes whose functions have been experimentally validated in *P. aeruginosa*, class 2 genes are highly similar to genes whose functions have been validated in other organisms, class 3 genes have hypothesized functions based on limited similarity to other genes or structural/functional domains, and class 4 genes are ORFs of unknown function. For the PA14-specific regions, 63% of the predicted ORFs have no known function, whereas only 28.9% of genes in the whole genome have class 4 annotations (Table 1). Therefore, gene identity alone could not indicate whether virulence-related genes were enriched in PA14-specific regions.

### Conservation of PA14-specific genes and their putative role in virulence

If a PA14 strain-specific region were functioning as a canonical pathogenicity island, then it should be more prevalent among other pathogenic isolates (and less prevalent among avirulent isolates). To test this hypothesis we needed to establish an objective measure of pathogenicity to compare different *P. aeruginosa *strains as well as to develop a high throughput method for determining the genomic content of the different strains. We used a model host infection system (the nematode *Caenorhabditis elegans*) to rank order the virulence of 18 diverse *P. aeruginosa *strains, using PA14 and PAO1 as reference strains. The 18 *P. aeruginosa *strains included 13 clinical isolates from a variety of infection types (CF lung infections, urinary tract infections, ocular infections, and blood isolates), one laboratory strain, and four environmental isolates. This same set of strains had been used in a previous study of *P. aeruginosa *strain diversity [9]. We used a custom microarray-based system (described below) to determine relatedness of the 20 (18 plus PAO1 and PA14) *P. aeruginosa *strains.

**Figure 4:**
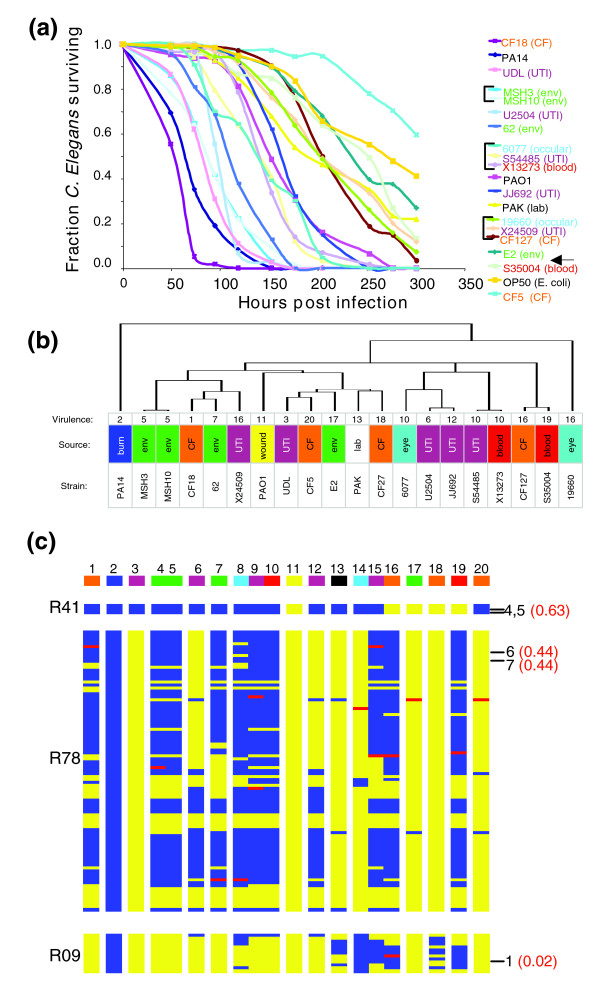
Poor correlation between PA14-pathogenicity genes and virulence in other strains. **(a) ***C. elegans *survival curves in the presence of 19 *P. aeruginosa *strains and OP50 (an *E. coli *control). The names of each strain tested are sorted according to the rank order of virulence, from most virulent at the top to the least virulent at the bottom, as determined by examining the time required to kill 50% of the nematodes. Black brackets indicate strains with indistinguishable virulence. Strain names are followed by the strain source and color-coded by strain source, as shown in panel b. Strain CF27 is not shown in this dataset; however, a similar experiment places its rank order virulence between strains E2 and S36004 (indicated by a black arrow). The same relative rank orders were obtained in two additional experiments. **(b) **Dendrogram representing the relatedness of 20 *P. aeruginosa *strains based on the presence or absence of genes as assayed by genomic DNA hybridizations to a custom microarray. Hierarchical clustering analysis was performed using the city-block distance metric. The name of each strain is shown, along with the source of the strain (UTI, urinary tract infection; CF, cystic fibrosis respiratory infection; env, environmental isolate), and the rank order virulence of each strain as determined in panel a (1, most virulent; 20, least virulent). Strains with indistinguishable virulence were given a tied rank order (also see Table 2). **(c) **Presence or absence of PA14 virulence genes in additional isolates. Data for each strain tested is presented in columns. Strains are arranged in order from left to right in order of decreasing virulence, in the same order as shown in panel a; column headers refer to the 20 strain numbers used in Table 2. Columns with no gaps between them represent groups of strains with indistinguishable virulence (strains 4 and 5; strains 8, 9 and 10; and strains 14, 15 and 16). Genes assayed are represented as rows, with adjacent rows representing ORFs present within a given gene cluster (PA14 region names shown on the left are abbreviated to remove the 'PA14' prefix). Each gene is described as present (blue), absent (yellow), or indeterminate (red). The positions of mutations resulting in reduced virulence in PA14 are indicated by lines to the right; numbers in black refer to entries in Table 3, and red numbers in parenthesis indicate the Spearman's rank correlation coefficients between presence or absence of the gene in other strains and their rank order virulence ratings.

In the *C. elegans *model pathogenicity system [13], an age-synchronized population of nematodes is fed the pathogen to be tested (instead of *Escherichia coli*, its traditional laboratory food source) and the longevity of the nematodes is determined. As shown previously, the longevity of *C. elegans *feeding on a particular *P. aeruginosa *strain correlates with virulence of the strain in mice [13,15,18]. The set of *P. aeruginosa *strains tested exhibited a full range of virulence phenotypes, including both the upper and lower limits that the assay system is capable of measuring (Figure 4a). PA14 (dark blue diamonds, second curve from the left) is extremely efficient at killing nematodes, whereas the less pathogenic PAO1 is intermediate (pink squares), killing more slowly than PA14 but more quickly than *E. coli *(negative control; yellow squares, second curve from the right).

The observation that PAO1 is more virulent than many other tested strains suggests that it has not become attenuated because of extensive passaging in the laboratory; rather, its virulence is probably that of a moderately pathogenic strain, with other strains more representative of truly 'avirulent' isolates. When comparing strains derived from the same type of infection, there was no consistent clustering with respect to their phenotype in *C. elegans*. For example, both the most and the least virulent strains tested were isolates from CF infections. Similarly, the five urinary tract infection strains exhibited a wide range of virulent to avirulent phenotypes. Importantly, two closely related environmental isolates (MSH3 and MSH10) were the fourth and fifth most virulent strains tested, indicating that nonclinically isolated strains can also have the potential to be infectious.

To test the hypothesis that the virulence of the 20 *P. aeruginosa *strains correlates with the presence of particular virulence islands, we performed a microarray-based analysis of the genomic content (a process described as genomotyping [23]) of the *P. aeruginosa *strains. We arrayed 285 synthetic oligonucleotides (70 mers) corresponding to PA14 genes that are absent in PAO1 and 130 oligonucleotides corresponding to PAO1 genes that are absent in PA14, along with additional sequences serving as positive and negative controls (see Materials and methods, below, and Additional data files 5 and 6). We first used the array data to generate a hierarchic clustering dendrogram showing the relatedness of the 20 *P. aeruginosa *strains (Figure 4b). Next, we used the *C. elegans *virulence data in Figure 4a to rank order the 20 strains and then examine the microarray data to determine whether the presence or absence of PA14-specific (or PAO1-specific) genes correlated with virulence (also see Table 2 and Additional data file 6). Strains were ordered from the most virulent (rank order 1) to the least virulent (rank order 20), with strains that had equivalent virulence assigned a tied rank. When the virulence rank order of each strain was superimposed on the dendrogram describing the relatedness of each strain based on genomic content (Figure 4b), no strong correlation was observed with the relative virulence of each strain in *C. elegans*. There are small clusters of similarly avirulent or virulent strains; for example, strains 6077, U2504, JJ692, S54485, and X13273 grouped together and possess similar virulence rankings (10, 6, 12, 10, and 10, respectively). However, these small clusters were occasionally punctuated by exceptions: a cluster of weakly virulent or avirulent strains (CF5, E2, PAK and CF27, with rank orders of 20, 17, 13, and 18, respectively) also includes strain UDL, which is the third most pathogenic strain tested.

In a previous study, Wolfgang and coworkers [9] used Affymetrix GeneChips to survey these same 18 strains (with the exception of PA14) for the presence or absence of PAO1 genes and found no obvious pattern that would correlate genomic content with disease (or the type of infection from which the isolate was derived). Similarly, examination of the distribution of PA14-specific genes among the tested isolates did not reveal any obvious clustering of genomic content with respect to the source of the strain (see dendrogram in Figure 4b).

### Identification of PA14-specific virulence genes and their conservation in other strains

The experiments described above showed that there was no correlation between the PA14-specific sequences in general and virulence in the *C. elegans *killing assay. We therefore performed a functional analysis of PA14-specific ORFs, identifying genes that were specifically required for pathogenicity and subsequently assessing their distribution among the other strains. Our laboratory has constructed a genome-wide, nonredundant transposon insertion mutant library in PA14 [24,25]. Using this library, we conducted a screen for mutants in PA14-specific genes that had reduced virulence in *C. elegans*. Nine genes were identified, which fell into six distinct clusters (Table 3). Three of these clusters are present in all of the other 19 *P. aeruginosa *strains but contain highly divergent sequences, including the O-antigen biosynthetic cluster (region PA14R38) and two groups of type 4 fimbrial biogenesis genes (regions PA14R77 and PA14R79). Oligonucleotides corresponding to the PA14-version of the O-antigen biosynthesis genes were included on the array and demonstrated that none of the other strains contain the PA14-versions of these genes; the genes for the two fimbrial synthesis clusters were not included on the array (because the sequences were not sufficiently divergent to meet our criteria for oligonucleotide design as outlined in Additional data file 1).

The remaining three clusters contain genes that are absent in PAO1. As indicated in Table 3, these three clusters contain five virulence genes, including one gene with putative transcriptional regulator activity and four ORFs of unknown function. Figure 4c summarizes the array data for these three regions. Each of the 20 strains tested are represented in columns arranged from left to right in order of decreasing pathogenicity (see Table 2 for rank order of virulence). The PA14 genes tested (shown in rows) are described as present (blue), absent (yellow), or indeterminate (red, indicating that the hybridization intensity was intermediate and a present or absent call could not be made with confidence; see Additional data file 1 for further details). The positions of the genes that result in an avirulent phenotype when mutated in PA14 are indicated on the right (also see Table 3). If a cluster of genes was required for or predictive of virulence in nematodes then we would expect to see a bias for present (blue) calls toward the left and absent (yellow) calls toward the right.

For these five virulence genes (and their associated three clusters), Spearman's rank correlation coefficients (relating their presence, absence, or indeterminate status with the virulence of the strain) were found to vary dramatically. The best correlation was found for two genes in region PA14R41 (correlation coefficients of 0.63). This is a cluster of eight genes known as the clone C-specific region common to clone C isolates (members of a clone family associated with CF infections [26]). Intermediate to no correlations were observed for the remaining two regions, namely PA14R78 and PAR09, which had correlation coefficients of 0.44 and 0.02, respectively. Of note, PA14R78 is a previously described pathogenicity island (PAPI-1) shown to contain genes required for pathogenicity in plants and mammals [19], although the two mutations in genes of unknown function that we identified in this cluster (PA14_59010 and PA14_59070; positions 6 and 7 in Figure 4c) were not among those previously examined. Regardless of the magnitude of the correlation, each virulence gene had exceptions to the expected trend, being present in attenuated strains and/or absent in virulent strains. Taken together, the functionally defined PA14 genes required for *C. elegans *killing are neither required for nor necessarily predictive of another strain's ability to be pathogenic.

## Conclusion

In this study we combined a traditional comparative genomic analysis with functional analyses, including genomotyping of other isolates, use of a genome-wide mutant library, and a model host infection system amenable to high-throughput screens, to ascertain the relationship between genomic content and virulence. We have found that PA14 and PAO1 are remarkably similar in total content and are largely colinear (with the exception of a previously described inversion). Because PA14 is significantly more virulent than PAO1 in most hosts, and previous studies have identified PA14 pathogenicity genes in regions that are absent in PAO1, we extended these observations to examine all of the PA14-specific genes for potential contributions to virulence. We began by examining 18 additional diverse isolates to assess the conservation of PA14-specific genes in other strains and whether they correlated with pathogenicity. In general, there was no obvious relationship between the presence of PA14-specific or PAO1-specific genes in other isolates and their virulence in *C. elegans *or the source of the strain. A specific examination of genes in these PA14-specific regions that were experimentally shown to contribute to virulence also failed to show a strong correlation between the presence of these genes and the pathogenicity of other isolates.

Our results amend the general view of pathogenicity islands, in which the acquisition of an island leads to the addition of gene products whose contribution to virulence is apparent based on the known function of the virulence gene and does not depend (necessarily) on genes outside of the island. For example, an island may contain a complete complement of genes required for the synthesis of secreted toxins, adhesins, invasion systems, iron uptake systems, or secretion systems (such as the type III and type IV systems) [1,2]. In these 'classic' cases, the presence or absence of individual pathogenicity islands correlates directly with a given gene product or process known to be required for virulence (and, therefore, with overall virulence). *P. aeruginosa *strains contain genes that fall into this category; for example, the presence of a pathogenicity island containing the type III secretion effector ExoU (present on PA14 region PA14R72) makes strains more cytotoxic to mammalian cells [27] and is required for pathogenicity in *Galleria *and the amoeba *Dictyostelium discoideum *[16,28]. However, we have also identified PA14 pathogenicity-related genes whose presence or absence does not correlate directly with degree of virulence, suggesting that these genes do not function autonomously to affect virulence. Our genomic analysis of PA14 virulence has demonstrated that pathogenicity in this organism is both multifactorial and combinatorial. Within a given isolate, virulence is multifactorial in that several factors combine to result in an overall virulence phenotype. Additionally, when comparing different strains, virulence is combinatorial in that pathogenicity factors may behave differently and that distinct combinations or groupings of these determinants may result in comparable virulence phenotypes.

What might account for the apparent complexity of PA14-specific virulence factors with respect to their conservation and role in other strains? First, it is clear that our analysis of genomic content in other strains is only an initial step in addressing similarities or differences in gene function among isolates; the apparent absence of a gene cannot exclude the existence of a functionally similar gene with significant sequence divergence, and the apparent presence of a gene cannot determine whether a gene is expressed. Transcriptional profiling of strain-specific genes (particularly in the presence of a putative host) will be a critical next step in clarifying the genes that contribute to virulence. Additional sequence information from other strains will be required to determine whether other functionally similar genes exist or whether orthologous genes are likely to be fully functional (intact ORFs with no polymorphisms that might alter protein function). Second, given the multifactorial and combinatorial nature of *P. aeruginosa *virulence, a full understanding of pathogenicity will require elucidation of how strain-specific genes (potentially responsible for differences in severity of disease among isolates) interact with core genome genes required for a base level of virulence. Our laboratory is currently extending the screen for PA14 mutants attenuated in *C. elegans *killing beyond the PA14-specific genes to include the entire genome (Liberati NT, Feinbaum RL, Ausubel FM, unpublished observations). Third, an important future direction will be to determine how generalizable our observations are in other model hosts. We are currently conducting pilot experiments to assess the viability of screening both the set of 20 *P. aeruginosa *strains and the PA14 mutant library in insects and plants to determine whether the presence or absence of identified PA14 virulence genes in other isolates correlates with their overall pathogenicity. A thorough understanding of networks of genes that are necessary for virulence in many or the majority of *P. aeruginosa *isolates (as opposed to genes that contribute to pathogenicity only in a subset of strains in which they are present) will be crucial for the design of effective therapeutics to combat the wide variety of human infections observed in clinical settings.

The evolution of virulence for a dedicated human pathogen generally involves the acquisition of discrete virulence functions required for specific interactions with the host, followed by gene loss related to specialization and potential restriction to the new environmental niche. In contrast, ubiquitous environmental micro-organisms continuously encounter dramatic changes in their ecosystem and the maintenance of genome complexity is preferable to optimization for a single niche. For environmental pathogenic fungi such as *Cryptococcus neoformans *that do not require animal hosts for replication or survival, the phenomenon of 'ready made' virulence has been described in which the selection and maintenance of virulence factors occurs during infection of environmental predators such as nematodes and ameba [29-31]. We propose that this multi-host view of pathogenic evolution also applies to environmental bacteria such as *P. aeruginosa *that infects nematodes, insects, plants, and ameba in the laboratory and probably encounter a similar range of potential hosts in the wild. The recent discovery that *C. elegans *pre-exposed to PA14 can modify its olfactory preferences to avoid the pathogen, and that this 'learned' avoidance behavior does not occur with nonpathogenic mutants of PA14, suggests that an interaction between these two organisms is biologically relevant [32]. Therefore, selection for pathogenicity may occur constantly in the environment; indeed, among the 20 isolates surveyed in this study, two environmental strains (MSH3 and MSH10) were among the most virulent, demonstrating that pathogenic potential exists even in nonclinical isolates. Furthermore, as an environmental organism, *P. aeruginosa *is not likely to undergo genome reduction associated with host restriction as in dedicated human pathogens. A notable exception is the case of CF infections, in which a clonal population remains isolated in a defined environment over a long period of time and tends not to spread to other patients. Indeed, a recent comparison of an early and late CF isolate from the same patient (at 6 months and 8 years of age) revealed an accumulation of multiple mutations leading to gene inactivation or loss, including a large 188 kb deletion [33]. When these mutated genes were examined in early and late infections of 29 additional CF patients, most of the observed mutations arose relatively late in the infections.

Our laboratory and others have previously shown that aspects of PA14 pathogenicity are conserved in model hosts and in mammalian hosts; PA14 virulence factors have been shown to be required for disease in both model hosts and mammals [13,14,16,17,19,28,34], and components of the host defense response are conserved in model hosts and humans [34-36]. Although these observations are critical in validating the use of simple, genetically tractable organisms as surrogate hosts to study human disease, perhaps the more appropriate perspective is to view nematodes, insects, plants, and ameba as the relevant natural hosts in which the selection for and evolution of pathogenic traits occurs, and the ability to subsequently infect humans is a secondary effect of these interactions.

## Materials and methods

### Shotgun genome sequencing

Shotgun sequencing of PA14 was performed using 65,800 plasmids containing 2-4 kb fragments of genomic PA14 DNA, resulting in over 10-fold coverage. Sequence reads were assembled using the Phred, Phrap, and Consed tools [37-39]. Details of finishing methods are described in Additional data file 1.

A long-range PCR-based method was used to assess whether particular *P. aeruginosa *genomes contain a PA14-like or a PAO1-like arrangement of genome sequence between the two most distant copies of a large ribosomal RNA cluster (Figures 1 and 2a,b; also see Stover and coworkers [7]). Primer pairs used to amplify two products specific for the PA14 chromosomal arrangement are as follows: 807.LL_rev (CGAACTGGAGGAAGTCTTCG) + 2-5'_2 (CGAGGCTTTCGTCTATCCAG), and 837.LL (AACTGGTGGAGGGAGAAGGAT) + 803.RR.rev (TAGCCTTCAATTCCACCTGG). Primer combinations used to amplify two PAO1-specific products are as follows: 807.LL_rev + 803.RR.rev, and 837.LL + 2-5'_2. These diagnostic primer pairs were also used to survey 18 additional strains (Table 2) to determine whether the PA14 or the PAO1 orientation was more representative of the ancestral *P. aeruginosa *chromosome. Of the 18 strains tested, none generated a PAO1-specific amplification product. Using the primers for the PA14 arrangement, seven of the 18 strains yielded products with both primer pairs, nine of 18 had a strong PCR product with one of the two primer pairs and a weak product with the second primer pair, and two of 18 exhibited a weak product with one primer pair and no product with the second.

### Sequence analysis and gene annotation

In determining GC content for PA14 and PAO1, standard deviations were determined using a continuous sliding window of 1 kb, and comparing the GC content for these regions with that of the whole genome. GC skew analysis was performed (using the formula GC skew = [n(G) - n(C)]/[n(G) + n(C)], where n [i] is the number of nucleotides, applied to 1 kb segments of each genome) to identify the peak cumulative GC skew as the likely position of the replication terminus [3]. Global alignments of the PA14 and PAO1 genomes were performed using the MUMmer 3.0 software package [40] to identify strain-specific regions. Automated ORF predictions were made using a combination of the BLAST and Glimmer2 algorithms [41,42]. Each predicted ORF was assigned a PA14 LocusName, beginning with 'PA14_' followed by five numerals. ORFs were numbered starting with PA14_00010 (*dnaA*), increasing in increments of 10 to allow for future insertions of additional genes or functional RNAs. Further details of ORF prediction and annotation are provided in Additional data file 1.

### Microarray genomotyping of *P. aeruginosa *strains

70-mer oligonucleotides for microarrays were designed as described previously [43] for 285 PA14-specific sequences, 130 PAO1-specific sequences, 11 genes common to both strains (to serve as positive controls), and additional genes and negative controls (see Additional data file 1). Chromosomal DNA from PA14, PAO1 (the sequenced isolate), and 18 additional *P. aeruginosa *strains described previously [9] was digested with HaeIII and labeled with Cyanine-3 or Cyanine-5 using the MICROMAX ASAP labeling kit (part #MPS544001KT; PerkinElmer, Wellesley, MA, USA). Labeled samples were combined in random pairs and hybridized to the arrays. Observed intensities for replicate hybridizations for each strain were averaged and log2 ratios were computed to determine the presence/absence of each gene based on a cut-off determined independently for each sample (see Additional data file 1). For each gene, a Spearman's rank correlation coefficient was calculated to describe the relationship between the spectrum of present/absent calls and the determined rank order virulence in *C. elegans *(see below). Heirarchical clustering analysis of strain relationships was performed using Cluster 3.0 [44] and Java Treeview [45]. All microarray data are included in Additional data files 5 and 6 and have also been deposited in ArrayExpress (ArrayExpress E-MEXP-824).

### *C. elegans *pathogenicity assays

The 20 *P. aeruginosa *strains analyzed by genomotyping were cultured overnight in Luria broth (LB) and assayed in the *C. elegans *killing system as described previously [13], with the exception that assays were performed using *fer-15(b26)ts;fem-1(hc17) *temperature-sensitive sterile mutant nematodes. The assays were carried out at the restrictive temperature (25°C) to prevent progeny formation and to allow the experiments to continue long enough to examine less pathogenic strains (for which progeny would typically overwhelm the assay plate by the end of the experiment). Bacterial strains are rank ordered from most to least virulent based on the position of the LT_50 _(the time at which 50% lethality was observed) in Figure 4a. In cases in which the LT_50 _was overlapping, rank orders were represented as a tied value for the strains in question. Strain CF27 is not included in the data shown in Figure 4a; however, its rank order in a similar experiment places it between strains E2 and S35004. The relative rank orders shown in Figure 4a are consistent with those observed in two additional experiments; although the absolute LT_50 _values increased or decreased for given strains between experiments, the relative rank orders remained consistent.

To screen for avirulent PA14 mutants, we utilized a nonredundant transposon insertion library [25]. All available mutants in PA14-specific genes (349 in total) were grown in 150 ml of LB media in 0.5 ml 96-well Masterblocks (part #786261; Greiner, Monroe, NC, USA). Cultures were agitated at 225 rpm and incubated for 16 hours at 37°C. A volume of 10 μl of each culture was spotted onto slow killing agar in 2 wells of a six-well plate [13,18], allowed to grow at 37°C for 24 hours, and left at room temperature for an additional 24 hours. Five L4 stage N2 nematodes were transferred manually to each well, and the number and age of progeny were recorded after 4 days at 25°C. The 349 mutants in PA14-specific genes were screened on two separate occasions, and any that were highly attenuated in either screen or were attenuated in both screens were re-examined in a secondary screen as described using wild-type N2 nematodes [13]. Six mutants were tested for growth in minimal (M63) media and all were found to have wild-type growth curves (Table 3). These included four of the five mutants in PA14 genes completely absent in PAO1 (examined in Figure 4c).

## Additional data files

The following additional data are available with the online version of this paper. Additional data file 1 is a document summarizing supplementary methods. Additional data file 2 is a table of PA14 gene annotations: the first worksheet contains the data, and the second worksheet contains a detailed description of the contents of the table and serves as a legend for the first worksheet. Additional data file 3 is a table showing the percentage of PA14 and PAO1 genes corresponding to each of 28 functional categories (shown for both the whole genome and strain-specific genes as described in Additional data file 4): the first worksheet contains the data, and the second worksheet contains a detailed description of the contents of the table and serves as a legend for the first worksheet. Additional data file 4 is a table summarizing properties of PA14-specific and PAO1-specific regions: the first worksheet contains the data, and the second worksheet contains a detailed description of the contents of the table and serves as a legend for the first worksheet. Additional data file 5 is a table describing the properties of oligos found on the genotyping microarray: the first worksheet contains the data, and the second worksheet contains a detailed description of the contents of the table and serves as a legend for the first worksheet. Additional data file 6 is a table of the microarray genotyping data for the 20 strains examined in this study (normalized average data and log2 ratio of normalized averages): the first worksheet contains the data, and the second worksheet contains a detailed description of the contents of the table and serves as a legend for the first worksheet. Additional data file 7 contains the coordinates of regions in PA14 or PAO1 that are common to four sequenced *P. aeruginosa *strains (PA14, PAO1, and two CF isolates: 2192 and C3719) and therefore comprise a putative *P. aeruginosa *core genome: the first worksheet contains the data, and the second worksheet contains a detailed description of the contents of the table and serves as a legend for the first worksheet.

## Authors' contributions

DGL and FMA were responsible for strategic planning and managing the overall project. LL, GG, KM, and RK were responsible for overseeing the raw sequencing, finishing, and initial assembly of PA14. DGL, NTL, SM, JH, and MS contributed to the raw sequencing effort. DGL performed manual edits to the assembly and additional finishing reactions. JMU and GW developed bioinformatic tools to analyze the sequence data. JMU, GW, and DGL performed the bulk of the bioinformatic analyses. DGL, JMU, LTD, NTL, JH, ED, and LF contributed to the manual annotation of PA14 genes. DGL designed the oligonucleotide array, performed the microarray experiments, and DGL and JMU analyzed the microarray data. NTL and RF performed the *C. elegans *assays. DGL and FMA wrote the paper. All authors discussed the results and commented on the manuscript.

## Supplementary Material

Additional data file 1 Summary of supplementary methods. Click here for file

Additional data file 2 A table of PA14 gene annotations. Click here for file

Additional data file 3 The percentage of PA14 and PAO1 genes corresponding to each of 28 functional categories (shown for both the whole genome and strain-specific genes as described in Additional data file 4). Click here for file

Additional data file 4 Summary of properties of PA14-specific and PAO1-specific regions. Click here for file

Additional data file 5 Properties of oligos found on the genotyping microarray. Click here for file

Additional data file 6 Microarray genotyping data for the 20 strains examined in this study (normalized average data and log2 ratio of normalized averages). Click here for file

Additional data file 7 Coordinates of regions in PA14 or PAO1 that are common to four sequenced *P. aeruginosa *strains (PA14, PAO1, and two CF isolates: 2192 and C3719) and therefore comprise a putative *P. aeruginosa *core genome. Click here for file

## Figures and Tables

**Table 1:** PA14 and PAO1 genome comparisons

	PA14	PAO1
Genome size	6,537,648	6,264,403
GC content	66.3 ± 4.3%^a^	66.6 ± 3.9%^a^
Total number of genes	5973	5651
Class 1 genes^b^	8.6%	9.0%
Class 2 genes^b^	18.0%	20.0%
Class 3 genes^b^	44.5%	26.8%
Class 4 genes^b^	28.9%	44.2%
Number of strain-specific regions^c^	58	54
Total DNA in strain-specific regions	541,215 (8.3%)	261,426 (4.2%)
GC content (strain-specific regions)	59.60%	59.80%
Total number of genes (strain-specific regions)	478	234
Class 1 genes^b^	3.3%	9.8%
Class 2 genes^b^	4.0%	3.0%
Class 3 genes^b^	29.7%	30.8%
Class 4 genes^b^	63.0%	56.4%

^a^Standard deviation was calculated using a sliding 1 kb window (see Additional data file 1). ^b^Classes 1-4 refer to the confidence rating assigned to the predicted gene function, as previously described [7,22]. Class 1 genes are those whose function has been experimentally validated in *P. aeruginosa*. Class 2 genes are highly similar to genes whose functions have been validated in another organism. Class 3 genes have hypothesized functions based on limited similarity to other genes or structural/functional domains. Class 4 genes are open reading frames (ORFs) of unknown function. ^c^Strain-specific regions with at least one ORF.

**Table 2:** Relative virulence of *P. aeruginosa *strains in *C. elegans*

Strain number^a^	Strain name	Strain source^b^	Rank order of virulence in *C. elegans*^c^
1	CF18	CF	1
2	PA14	burn	2
3	UDL	UTI	3
4	MSH3	Environmental	5
5	MSH10	Environmental	5
6	U2504	UTI	6
7	62	Environmental	7
8	6077	Ocular	10
9	S54485	UTI	10
10	X13273	Blood	10
11	PAO1	Wound	11
12	JJ692	UTI	12
13	PAK	Laboratory	13
14	19660	Ocular	16
15	X24509	UTI	16
16	CF127	CF	16
17	E2	Environmental	17
18	CF27	CF	18^d^
19	S35004	Blood	19
20	CF5	CF	20

^a^This strain number corresponds to the order in which strains are listed in Figure 4c (microarray) from left to right. ^b^References for each strain are listed in the report by Wolfgang and coworkers [9]. ^c^Identical rank orders are shown for two or more strains with indistinguishable virulence. ^d^Strain CF27 is not included in Figure 4a; its relative order is inferred based on other experiments. CF, cystic fibrosis; UTI, urinary tract infection.

**Table 3:** PA14-specific regions containing genes required for *C. elegans *killing

PA14 region ID	Number of genes in region	GC content^a^	PA14 gene ID	Previously assigned gene name	Gene function	Correlation coefficient of gene with rank order of virulence	Position of gene in Figure 4c
PA14R09	14	58.5	PA14_03370^b^		Unknown	0.02	1
PA14R38	12	46.4	PA14_23420^b^	ORF_10	O-antigen biosynthesis		2^c^
			PA14_23430	ORF_11	O-antigen biosynthesis		3^c^
PA14R41	8	66	PA14_27680^b^	C118	Unknown	0.63	4
			PA14_27700^b^	C120	Putative transcriptional regulator	0.63	5
PA14R77	3	53.9	PA14_58760	*pilC*	Type 4 fimbrial biogenesis		Not on oligo array^c^
PA14R78	112	59.7	PA14_59010^b^	RL107	Unknown	0.44	6
			PA14_59070	RL102	Unknown	0.44	7
PA14R79	2	54.9	PA14_60290^b^	*pilW*	Type 4 fimbrial biogenesis		Not on oligo array^c^

^a^GC content of the PA14 genome is 66.3 ± 4.3%. ^b^These mutants were grown on minimal media and all had wild-type growth curves. ^c^These gene clusters are present in all *P. aeruginosa *isolates, but the sequences are highly divergent. Oligos for the PA14-specific O-antigen cluster were included on the array, but none of the other strains contained the PA14-version of these genes. The two clusters involved in type 4 fimbrial biogenesis were not included on the array because the sequences were not sufficiently different to pass our criteria for oligo design (see Materials and methods).
