# Supplementary material for: Genomic analysis reveals that Pseudomonas aeruginosa virulence is combinatorial
Source: Genome Biol. 2006 Oct 12;7(10):R90. doi: 10.1186/gb-2006-7-10-r90 (PMC1794565; doi:10.1186/gb-2006-7-10-r90)
Supplement: Additional data file 1 — Summary of supplementary methods. [file gb-2006-7-10-r90-S1.doc]

**Ausubel Additional data file 1**

**Supplementary Methods**

**Shotgun Sequencing and Finishing**

Shotgun sequencing of PA14 was performed using 65,800 plasmids containing 2-4 kb fragments of genomic PA14 DNA, sheared by nebulization, end-repaired and cloned into the pSMART vector (Lucigen). Sequencing reactions were performed using ABI BigDye 3.1 dye terminator chemistry and ABI 3700 Capillary DNA Sequencers, and reads were assembled using the Phred, Phrap and Consed tools [37-39]. Three rounds of directed sequencing were performed that included re-sequencing of clones at the ends of contigs with altered nucleotide ratios and primer-walking reactions off the ends of contigs, followed by manual end-trimming of contigs allowing for additional joins and yielding 13 contigs with an estimated error rate of <0.26 per 10,000 bp. Scaffolding of these contigs on the existing PAO1 genome resulted in a predicted contig order, and PCR reactions were performed to isolate gap-spanning sequences. We also used annotations of PA14 ORFs (see below) and BLAST alignments to identify regions that contained putative frame-shifts (owing either to sequencing errors or bona fide sequence polymorphisms). Chromatograms for 113 regions were manually examined using Consed. In 32 cases, errors in consensus sequence assignments were identified and manually corrected to match the reading frame in PAO1. For the remaining cases in which manual inspection did not provide an obvious resolution, we identified 96 clones for re-sequencing in ambiguous regions. In total, 67 regions were corrected (either by manual editing or re-sequencing), 39 regions were not changed and confirmed to be sequence polymorphisms with PAO1, and 7 regions could not be re-sequenced and remain possible polymorphisms. For those genes in which the sequence was corrected, there was a relative over-representation of class 1 or 2 genes for which the gene function is known or likely to be known (39 / 67 cases = 58%). In contrast, in the 39 cases in which a sequence polymorphism was confirmed between PAO1 and PA14, the majority of cases involved class 4 genes (ORFs of unknown function, 28 / 39 cases = 72%).

**Sequence Analysis and Annotation**

Global alignments of the PA14 and PAO1 genomes were performed using the MUMmer 3.0 software package [40]. Regions that failed to align with the other genome (gaps in alignments defined using the NUCmer component of MUMmer) were identified as possible strain-specific regions (see Additional data file 4). Genes within these regions were inspected manually and defined as orthologs (90% or higher amino acid identity), polymorphisms (50 to 90% identity), or strain-specific (less than 50% identity). Additional classifications assigned to genes included translocations, gene duplications, fusions (when the gene resulted from the fusion of two adjacent ORFs in the other strain), or split ORFs (when two genes correspond to a single ORF in the other strain). Based on the identities of genes at the boundaries of strain-specific regions, the coordinates of the regions were sometimes expanded to include full open reading frames that had significant amino acid differences (enough to be considered polymorphisms) but not enough nucleotide sequence differences to be identified by NUCmer, and adjacent strain-specific regions were occasionally fused together into one larger region. Finally, for each strain-specific region in one strain, the corresponding region was determined in the other strain based on the common genes bordering the strain-specific regions. If one strain had no NUCmer detected gap in a locus occupied by a strain-specific region in the other strain (i.e. – the second strain contained a region that was an insertion in the genome of the first strain), then a single nucleotide position was identified in the first strain to serve as an approximate reference point for the position of the insertion. Each strain specific region was then given a name using the prefixes PA14R and PAO1R for PA14 and PAO1, respectively, followed by a two-digit number from 01 to 85 assigned to regions arranged in linear order in each genome starting from the origin of replication. For each region, Additional data file 4 lists the name of the corresponding region in the other strain, the start and stop coordinates, the length of the region, the total number of genes (some regions contain no genes, either because they are NUCmer-defined gaps in alignments that contain no predicted ORFs, or because the region has been manually defined as a single nucleotide reference point for the position of an ORF-containing region in the other strain), the breakdown of categories of genes in the region (strain-specific, polymorphism, ortholog, duplication, translocation, split ORFs), a breakdown of the gene product confidence code assignments for all genes in the region (class 1, 2, 3, and 4 genes), the total number of genes in each of the 28 functional categories used in the gene annotations, and the method used to define the region (NUCmer only, NUCmer combined with manual edits of the region boundaries, or manual edits only for defining regions missed by NUCmer).

Automated ORF predictions were made using a two-step process. A BLAST-based method was used to compare PA14 sequences with protein sequences from PAO1, *P. syringae* strain B728A, and predicted genes from two previously described PA14 pathogenicity islands [19], using a minimal cutoffs (bit score = 100 and percent identity = 40). For each successful BLAST match, a gene prediction was recorded only if putative start and stop positions could be identified within 60 bp of the analogous positions in the previously annotated genes and if no in-frame stop codons were detected. A second round of ORF predictions utilized Glimmer2 [41, 42]. An interpolated Markov model for gene prediction was generated using PAO1 genes as a training set. All annotations are available at the Ausubel lab PA14 sequencing website [20].

Each predicted ORF was assigned a PA14 LocusName, beginning with “PA14_” followed by five numerals. ORFs were numbered starting with PA14_00010 (dnaA), increasing in increments of 10 in order of the lesser of the start or the stop position of the gene as the genome was scanned from left to right. Increments of 10 were used to allow for future insertions of additional genes or functional RNAs. ORF annotations were performed using a web-based application developed in-house for collaborative manual entries and editing. Automated BLAST searches against GBBCT (the GenBank bacterial database), RPS BLAST, and Needleman-Wunsch global alignments of protein alignments were performed to assist the annotators. The initial set of predicted ORFs included many that were overlapping (on opposite strands, or on the same strand in different reading frames). Putative overlapping pseudogenes were identified and removed from the final list of annotations as follows: (1) ORFs overlapping by 50 or more bases were identified, (2) the gene with the higher confidence level for gene function assignment was kept, (3) in the event that both genes had equal confidence classes, if one gene had an assigned gene name (and the other did not), it was kept, and (4) all remaining overlaps were manually inspected. PA14 and PAO1 ORFs were compared to identify putative pairs of orthologs and strain-specific genes. PA14 genes were BLASTed, one at a time, against the collection of PAO1 genes and this process was repeated in reverse (i.e. – PAO1 genes BLASTed against the collection of PA14 genes). Best BLAST matches were determined based on BLAST scores and synteny with previous genes to maintain gene order. Reciprocal best BLAST matches were recorded as likely pairs of homologs. In cases where no match was obtained, ORFs were BLASTed against the whole genome of the other strain; BLAST matches in the nucleotide sequence of the other genome were examined to determine if an ORF existed but was not annotated, or if no ORF was present due to sequence polymorphisms. Genes that resulted in no BLAST match to both the genes and the genome of the other strain were recorded as putative strain-specific genes. Locations of reciprocal best blast matches were graphed (Figure 3) and found to correlate well with the global alignments made of the raw nucleotide sequences using the mummerplot component of the MUMmer 3.0 package.

**Microarray Genomotyping of *P. aeruginosa* Strains**

PA14-specific and PAO1-specific genes were identified by performing BLAST searches of all annotated ORFs in one strain against the genome of the other strain and selecting those that failed to meet minimal criteria for a positive match. These criteria included a minimal nucleotide percent identity of 95, a minimal amino acid percent identity of 70, and a maximal allowed difference between the length of the alignment and the full gene of 20 percent. 70-mer oligonucleotides were successfully designed as described [43] for 305 PA14-specific genes, 144 PAO1-specific genes, 14 genes common to both strains (to serve as positive controls), 11 genes diagnostic for different O-antigen biosynthetic clusters, and 13 negative controls. BLAST analysis confirmed that 70-mer oligos designed to be present in a given genome had a 68/70 bp match or better, and oligos designed to be absent in a genome had at best a 28/31 bp match. These oligos were arrayed in quadruplicate on microscope slides by the MGH Microarray Core Facility (Cambridge, MA).

Chromosomal DNA from PA14, PAO1 and 18 additional *P. aeruginosa* strains described previously [9] was prepared using the FastDNA kit (Bio101, part # 6540-400) according to the manufacturer’s recommendations for bacterial chromosomal DNA, and digested with HaeIII as described [47], and directly labeled using the MICROMAX ASAP labeling kit (PerkinElmer, part #MPS544001KT). Each sample was labeled at least one time with Cyanine-3 and one time with Cyanine-5 reagents. 2 mg of Cy-3 and Cy-5 labeled samples were combined in random pairs and hybridized to the arrays on a GeneTAC Hybridization Station. Arrays were scanned with an Axon GenePix 4000B scanner using the GenePix Pro 3.0 software, and preliminary data analysis was performed using the BioArray Software Environement (BASE) developed at the MGH Microarray Core Facility [48].

Examination of the 14 oligonucleotides corresponding to genes present in both PA14 and PAO1 revealed that 11 were present in all strains in single copy. These corresponded to the following oligonucleotide IDs (with gene IDs and gene names following in parentheses):

44463_533 (PA14_09400, phzS); 43176_372 (PA14_00050, gyrB); 44305_612 (PA14_42340, pscD); 45033_502 (PA14_17530, recA); 50003_839 (PA14_00560, exoT); 45111_21 (PA14_09490, phzM); 43445_739 (PA14_00640, phzH); 43335_1867 (PA14_07530, dnaG); 44411_1148 (PA14_09460, phzC1); 48825_496 (PA14_39945, phzC2); 46721_291 (PA14_30650, gacA).

Data for these 11 oligos were used to normalize the data for each channel (Cy-3 or Cy-5) on each slide to correct for differences in labeling or hybridization efficiencies. The average raw intensity values for these 11 oligos were identified for each sample, and divided by 20,000 (the approximate average intensity for these 11 oligos for all samples) to generate a scaling factor (SF). The SF was used to divide each of the individual intensity values for each sample to generate a set of scaled (or normalized) intensity values. Scaled intensities for replicates of each strain tested were averaged (either 2 or 3 experiments were averaged for each strain, except for PA14, for which 4 experiments were averaged). Averaged, scaled intensities were converted to log2 ratios by comparing each value to a reference. For PA14-specific genes, or control genes present in both PA14 and PAO1, the average scaled values for PA14 were used as the reference; for PAO1-specific genes, average scaled PAO1 values were used as the reference. We attempted to determine present or absent calls for each gene as described [23]; however, our data set was too small with respect to the total number of genes assayed for this method to be effective. Instead, the distribution of log2 ratio values was plotted for each strain (using a bin size of 0.25), and a roughly bimodal distribution was observed in all cases, with one peak of log2 ratios corresponding to genes present in the tested strain (generally centered between 0 and -1) and a second, broad peak corresponding to absent genes (with log2 ratios of approximately -1.5 and below). For each strain, a range was determined that lay in between the two peaks, for which the present or absent call would be classified as indeterminate. Log2 ratio values above this range resulted present calls, and values below this range were absent calls. The empirically determined ranges of indeterminate values are as follows: -1.2 to -0.8 for PA14 and PAO1; -1.4 to -1.0 for CF27; -1.7 to -1.3 for 62, 6077, 19660, CF5, MSH3, MSH10, S54485, U2504, UDL and X13273; -2.0 to -1.6 for CF18, CF127, E2, JJ692, and PAK; -2.2 to -1.8 fro X24509; -2.8 to -2.4 for S35004. Using these cut-off values, oligos were identified that failed to yield the expected results with respect to PA14 and/or PAO1 (i.e. – they were called present in PA14 when BLAST results predict that they should be absent). These oligonucleotides (4 PA14-specific oligos and 3 PAO1-specific oligos) were removed from the analysis. The properties of the oligonucleotides utilized in the final analysis are shown in Additional data file 5. Log2 ratio and raw data for all samples are shown in Additional data file 6.

For subsequent cluster analysis of the microarray data, present calls were converted to “3”, indeterminate to “2”, and absent calls to “1”. For each gene, a Spearman’s rank correlation coefficient was calculated to describe the relationship between the spectrum of present/absent calls and the determined rank order virulence in *C. elegans* (see below). Heirarchical clustering analysis of strain relationships was performed using Cluster 3.0 [44] and Java Treeview [45]. The city block distance metric is shown in Figure 4B. Other distance metrics (Euclidian, Spearman Rank Correlation, and Pearson Correlation Centered) were also used with similar results.
